# Supplementary material for: Isolation of a Marker Olean-12-en-28-butanol Derivative from Viscum continuum E. Mey. Ex Sprague and the Evaluation of Its Antioxidant and Antimicrobial Potentials
Source: Plants (Basel). 2024 May 16;13(10):1382. doi: 10.3390/plants13101382 (PMC11125446; doi:10.3390/plants13101382)
Supplement: Supplementary file 1 [file plants-13-01382-s001.zip › plants-2925448-supplementary.pdf]

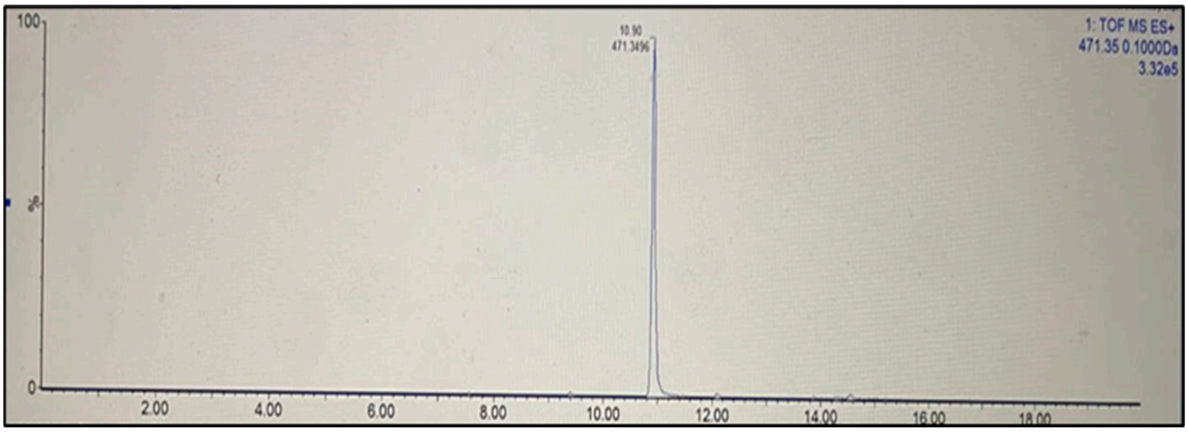

Figure S1. Total ion chromatogram of **D4** during analysis indicating the purity of the isolate as detected by diode array and TOF MS ESI-BPI.

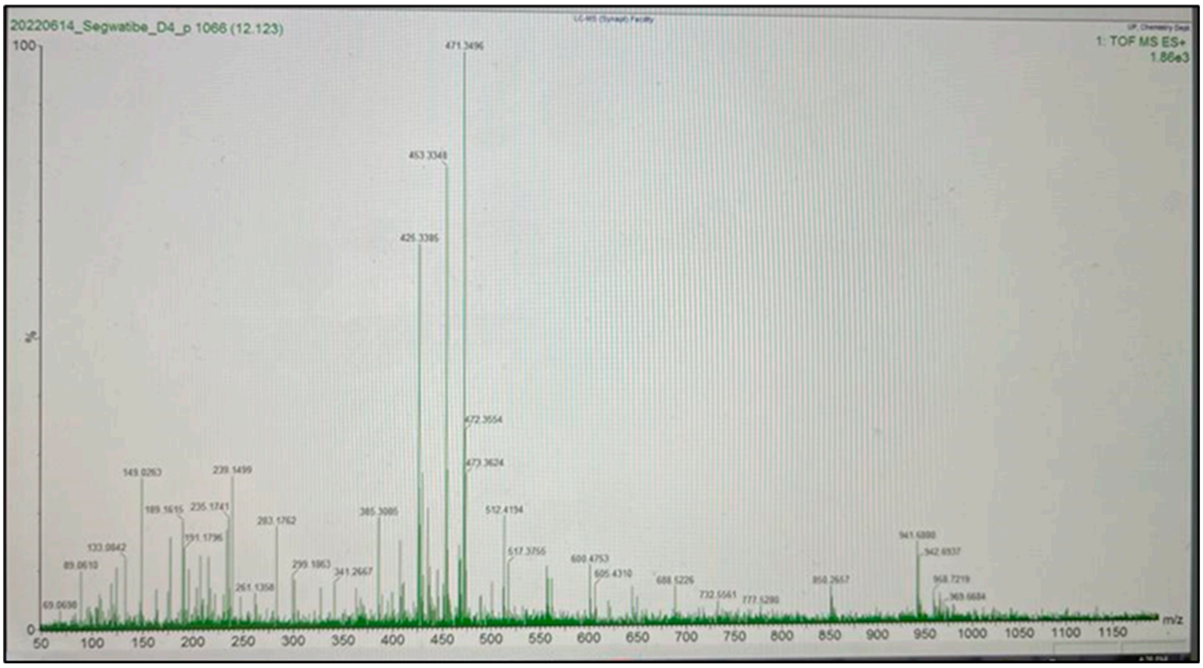

Figure S2. UPLC-TOF MS indicating the fragments of D4 (453, 426, 386, 283, 239, 149)

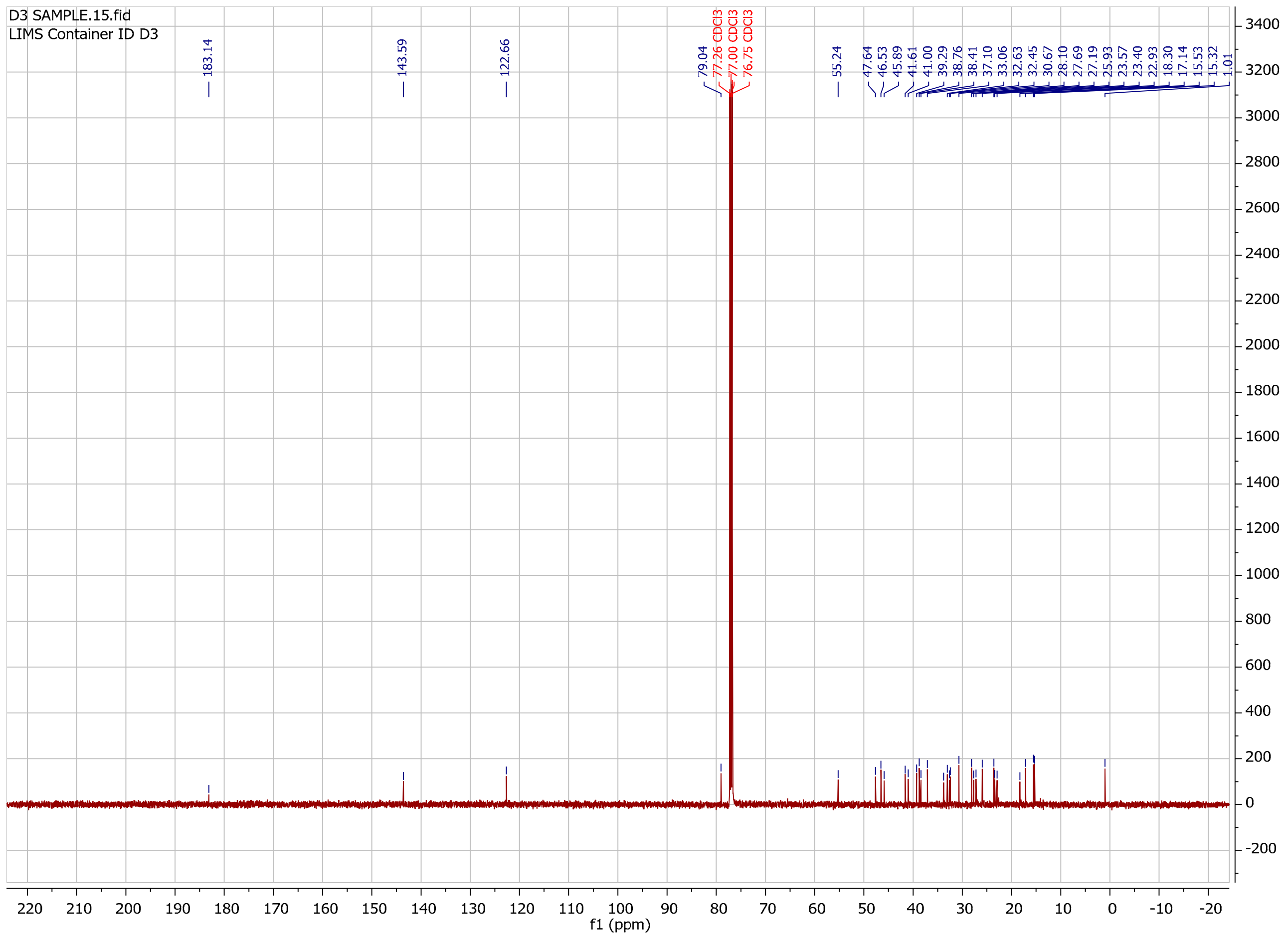

Figure S3. C-13 NMR of D4

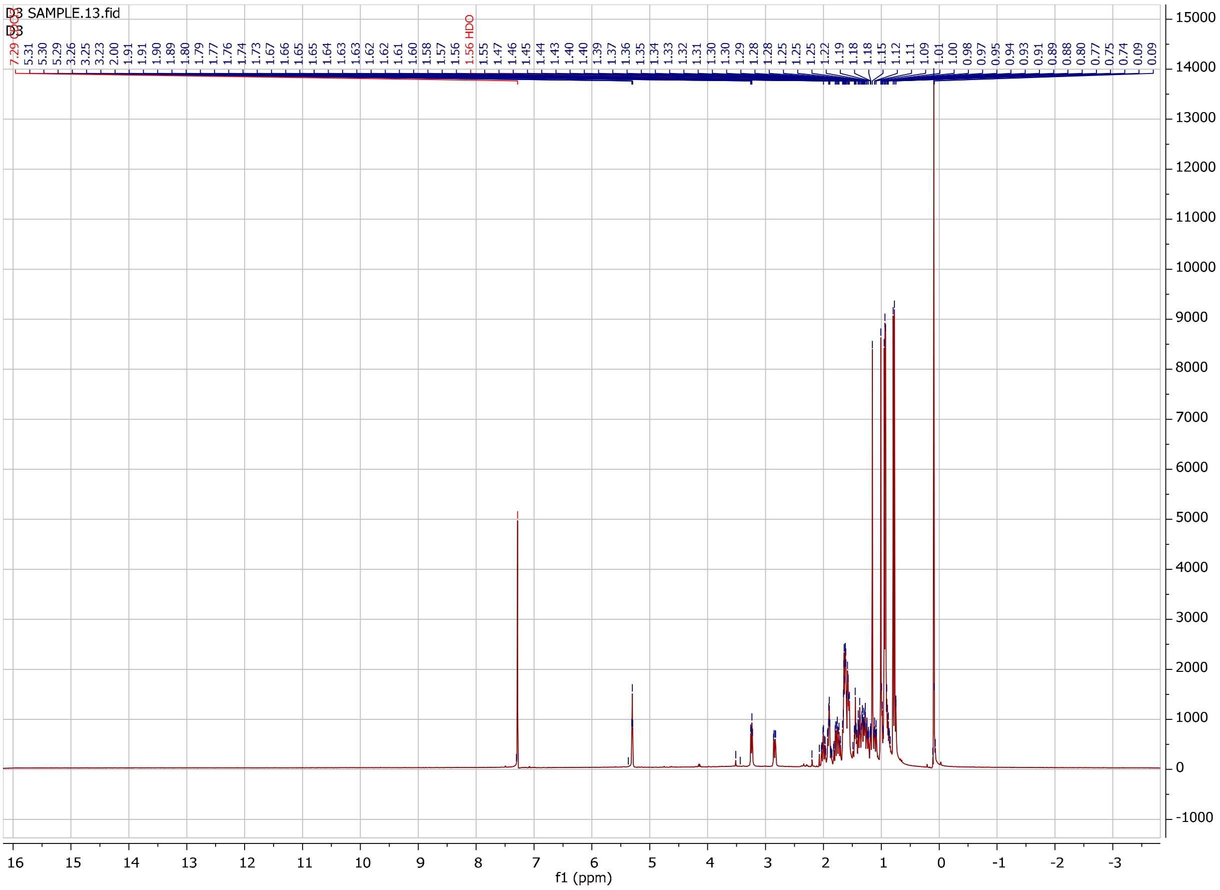

Figure S4. H-1 NMR of D4

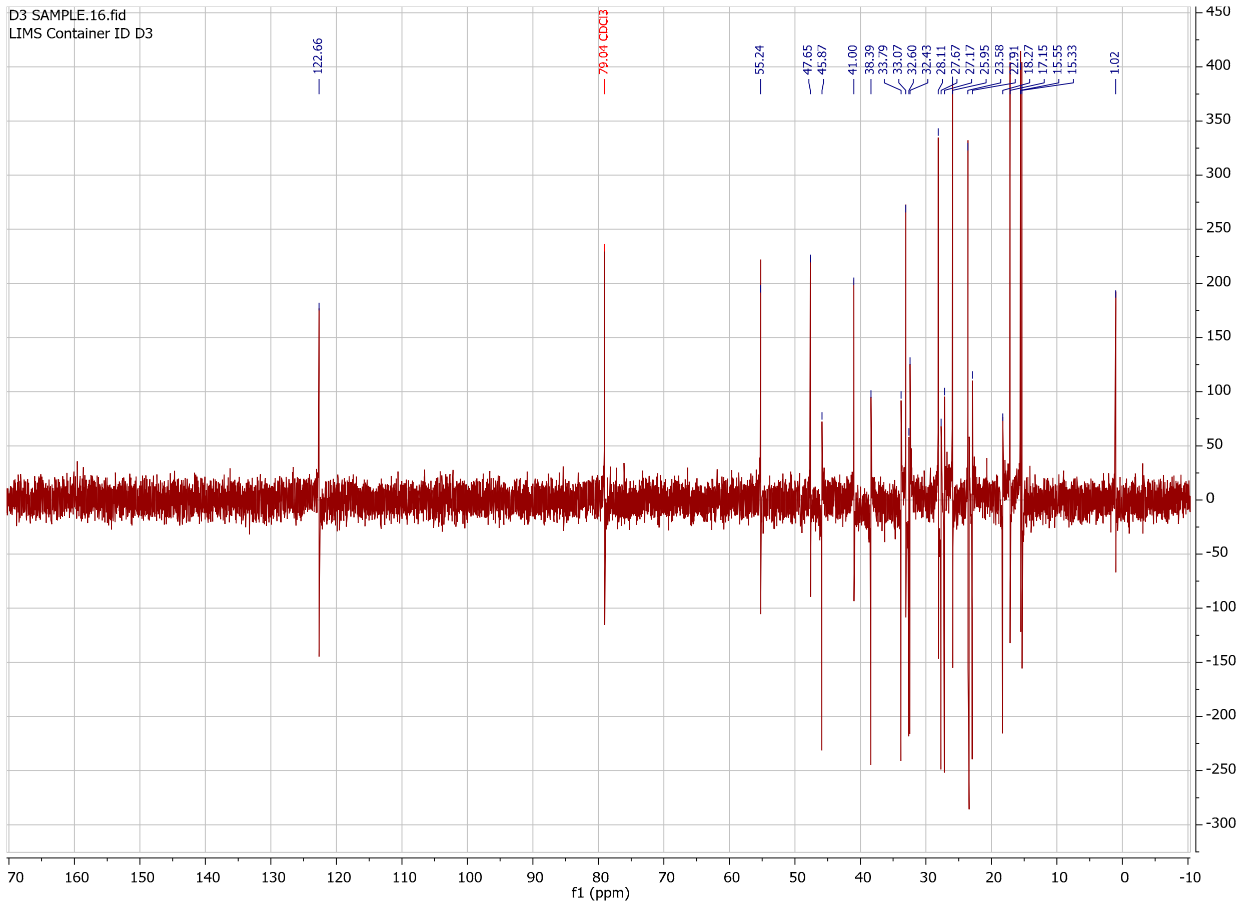

Figure S5. DEPT NMR of D4

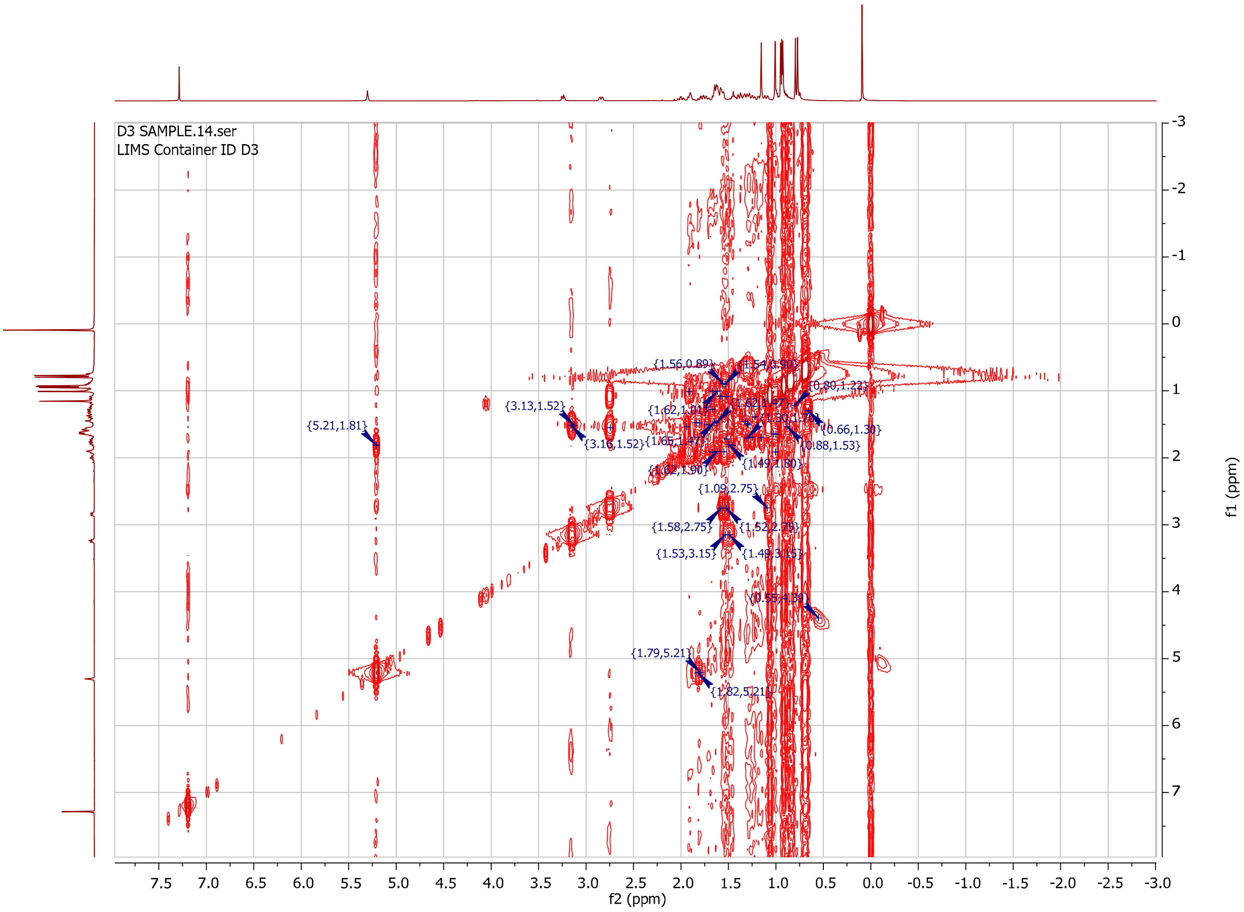

Figure S6. COSY NMR of D4

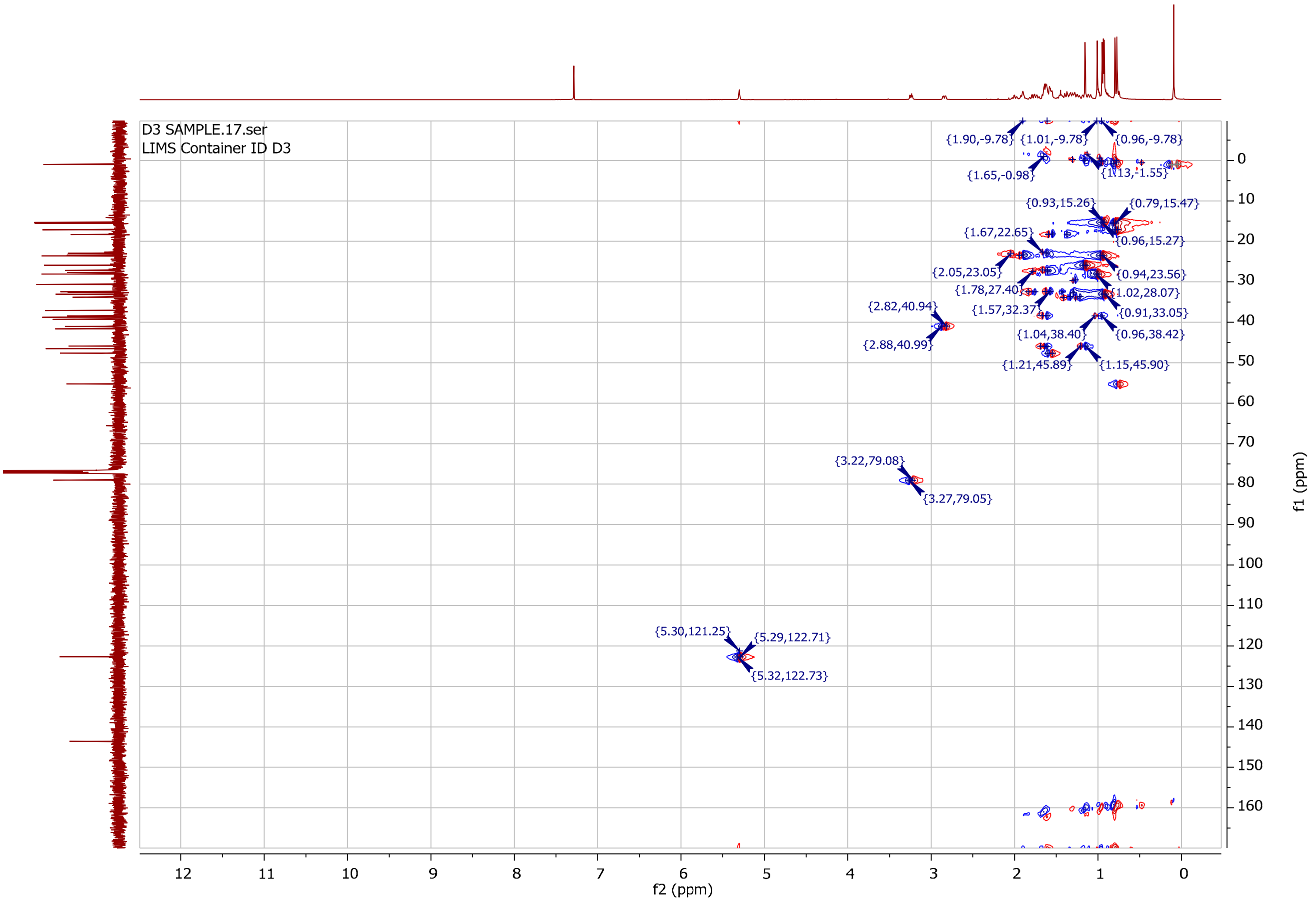

Figure S7. HSQC NMR of D4

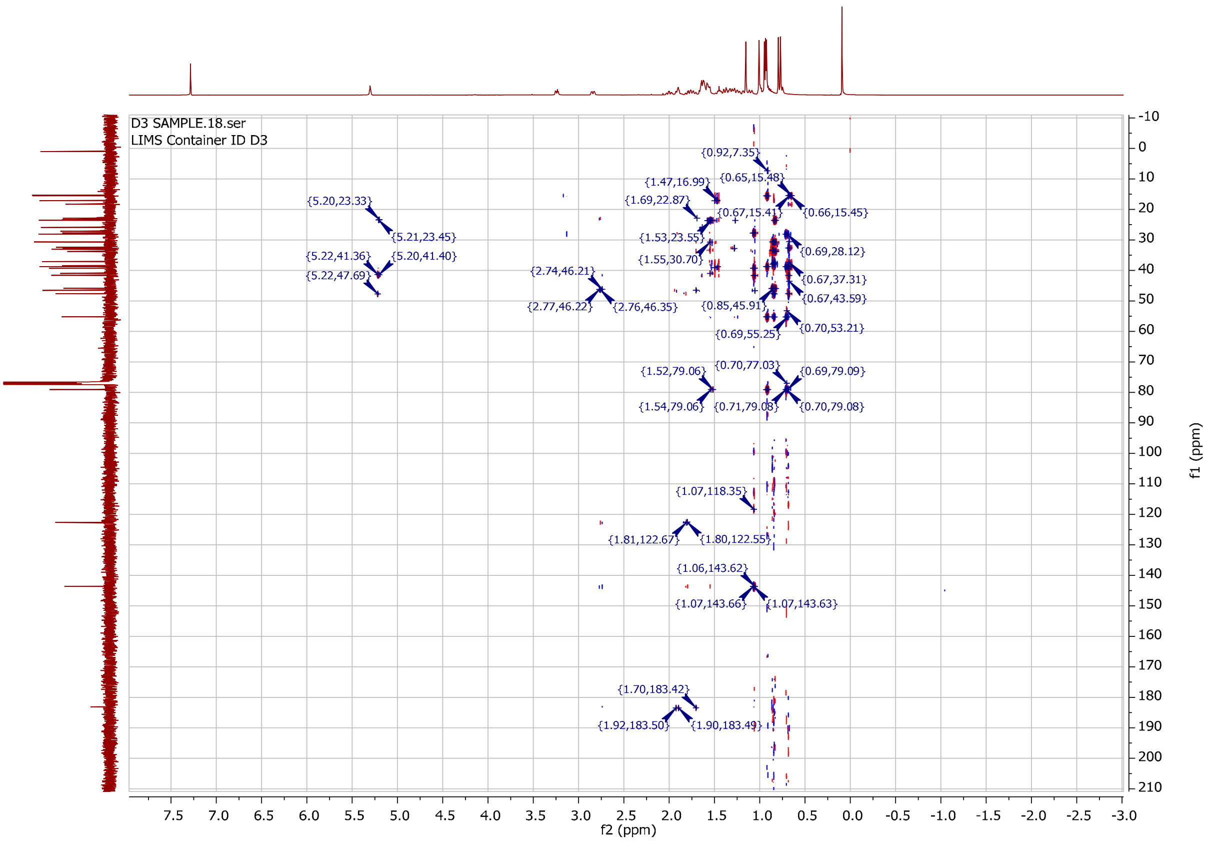

Figure S8. HMBC NMR of D4
